# *In vitro* antibacterial effects of silver nanoparticles synthesized using *Verbena officinalis* leaf extract on *Yersinia ruckeri, Vibrio cholera* and *Listeria monocytogenes*

**Published:** 2018-12

**Authors:** Narjes Sanchooli, Saeide Saeidi, Hashem Khandan Barani, Esmael Sanchooli

**Affiliations:** 1 Department of Fisheries of Hamoun International Wetland Research Institute, University of Zabol, Zabol, Iran; 2 Zabol Medicinal Plant Research Center, Zabol University of Medical Sciences, Zabol, Iran; 3 Department of Chemistry, University of Zabol, Zabol, Iran

**Keywords:** Antibacterial activity, Green synthesis, *Verbena officinalis*, Minimum inhibitory concentration

## Abstract

**Background and Objectives:**

The use of plants for the synthesis of nanoparticles has received attention. The present study aimed to evaluate the antibacterial effects of silver nanoparticles synthesized by *Verbena officinalis* leaf extract against *Yersinia ruckeri, Vibrio cholerae* and *Listeria monocytogenes*.

**Materials and Methods:**

Silver nanoparticles were obtained by reacting silver nitrate solution 2 mM and *V. officinalis* leaf extract. The AgNPs were characterized by UV-visible spectrophotometer, scanning electron microscopy (SEM), and Fourier transform infrared spectrometer (FTIR). To determine minimum inhibitory concentration and test antibiogram of nanoparticle synthesized, broth micro dilution and agar well diffusion methods were used, respectively.

**Results:**

The zones of bacterial inhibition were 16 ± 0.5 and 9.16 ± 0.28 mm against *Y. ruckeri* and *L. monocytogenes* using 10 and 0.62 mg/mL AgNPs, respectively. Among the studied bacterial species, silver nanoparticles were more effective on *Y. ruckeri* and *L. monocytogenes* and less effective on *V. cholerae*. The highest MIC and MBC of AgNPs (2.5 and 5 mg/mL) were observed for *V. cholerae*. The lowest MIC and MBC of AgNPs (0.32 and 0.62 mg/mL) were observed for *Y. ruckeri*, respectively. The MIC and MBC of AgNPs were found to be 1.25 and 2.5 mg/mL for *L. monocytogenes*.

**Conclusion:**

The results clearly indicated that *V. officinalis* AgNPs have potential antimicrobial activity against Gram-positive and Gram-negative bacteria.

## INTRODUCTION

Nanotechnology is a general term that refers to all advanced technologies in the field of working with nanoscale. Usually the purpose of nanoscale dimension is about 1 nm to 100 nm (1). Nano-materials based on metal ions have wide cytotoxicity activity against bacteria, fungi and viruses. Nano materials and specially, nano-metal materials, due to having a superficial load and the ratio of surface to volume, disable enzymes and DNA of microorganisms with electron balance between groups of electron donor, such as, thiol, carboxylate, amide, imidazole, indole and hydroxyl (2). Various methods are used for producing nanoparticles, such as reactions of chemicals and photochemicals in reverse micelles, thermal analysis compounds with the help of radiation, electrochemical methods, sonochemical, processing with microwaves. Unfortunately, the use of hazardous substances is inevitable to produce nanoparticles in most methods (3). The disadvantages of synthesis methods of nanoparticle are the use of harmful toxic chemicals, low conversion rates, high consumption of materials and energy. Thus, for overcome these limitations, the growing demand for the development of safe and environment-friendly process of the synthesis of nanoparticles is required.

Green synthesis techniques or biological methods that use microorganisms, such as fungi, bacteria, or plant extracts, are an alternative method of simple and practical for physical and chemical methods. The use of plant extracts for biological synthesis of nanoparticles is an important method. It is rapid, cost-effective, environment-friendly and has received attention (4). So far, the biosynthesis of silver nanoparticles by plants, such as *Artemisia nilagirica* (5), *Piper longum* (6), *Acalypha indica* (7), and many others, has been conducted. *Verbena officinalis* is perennial herb native to Europe. It is used in folk medicine for the treatment of inflammatory disorders (8, 9), gastric diseases, abrasions, and skin burns (10, 11). It is commonly used for biological activity, pharmacokinetic effect, antitumor, antioxidant and antifungal activity, antiradical efficacy, analgesic activity, neuroprotective effects, anti-inflammatory activity, and antioxidant activities (12). A recent survey indicated that the main ingredients in the essential oil of *V. officinalis* are citral, geranial, neral, geraniol, limonene and cineole (13). The 3 bacteria studied were selected because *Yersinia ruckeri* is the causative agent of enteric red mouth disease, especially rainbow trout and atlantic salmon fish (14). Infections due to *Y. ruckeri* cause high mortality in fish aquaculture systems, leading to significant economic losses in the fish farming industry (15). The bacterium is shed in the faeces of infected fish and the disease can be transmitted by water. *Listeria monocytogenes* is a Gram-positive foodborne pathogen that is ubiquitously found in diverse environments, such as soil, water, various food products, animals and humans (16), which is often transmitted through contaminated water and food (17). *Vibrio cholerae* is a facultative human pathogen found in coastal waters and causes acute gastrointestinal disease, cholera, a major health threat in poor nations (18). *V. cholerae* causes severe diarrhea in humans (19).

In this study, *V. officinalis* leaf extract was used to convert the silver ions to silver nanoparticles by silver nitrate solution. The antibacterial effects of silver nanoparticles synthesized by *V. officinalis* leaf extract was evaluted against *Y. ruckeri, V. cholerae* and *L. monocytogenes*.

## MATERIALS AND METHODS

### Preparation of material and microorganisms.

Leaf of *V. officinalis* were obtianed from the farms growing medicinal plants at the agricultural research institute located at Baghiatoallah Azam Complex of University of Zabol in Zabol. AgNO _3_ and Nutrient agar (NA) were purchased from Merck Company. The microorganisms used in this experiment were *Listeria monocytogenes* (1298), *Yersinia ruckeri* (KC291153), and *Vibrio cholerae* (1611).

### Preparing plant extracts and synthesis of silve nanoparticle.

To prepare aqueous extracts of *V. officinalis*, first, leaves of *V. officinalis* were washed 2 times, once with purified water and once with distilled water. The leaves were air-dried under shade and powdered using a disintegrator. About 2 grams of the sample was added to 20 mL sterilized distilled water and was placed in a shaker incubator for 2 hours. The extract was cooled and filtered using Whatman no1 filter paper (20). For the synthesis of silver nanoparticle, the stocked silver nitrate solution 0.1 M (0.169 g in 10 mL of distilled water) was prepared. Synthesis of silver nanoparticles was done by mixing and reaction of silver nitrate solution of 2 mM and extract of *V. officinalis* (1 mL of the extract was obtained by 19 mL distilled water and combined; then, 400 μL from silver nitrate solution of 0.1 M was added). Next, the mixture was placed in the incubator at a temperature of 37°C for 24 hours.

### Characterization of silver nanoparticles, UV-Vis spectra analysis.

Sample (1 mL) of the suspension was collected periodically to monitor the completion of bioreduction of Ag+ in aqueous solution, 1 mL of sample was diluted with 2 mL of distilled water; then, the UV-Vis spectrum of solution was measured between wavelength 280 to 700 nm in a spectrophotometer (Rayleigh, UV-2100, China), with a resolution of 1 nm.

### Fourier transform infrared spectroscopy analysis.

The dried Ag NPs were analyzed using the potassium bromide (KBr) pellet (FTIR grade) method in 1:100 ratio and the spectrum was recorded using FTIR (Bruker optics Ft Tensor, 27, Germany).

### Scanning electron microscopy analysis.

AgNPs was centrifuged at 10000 rpm for 15 minutes. Supernatant was collected and poured nanoparticles were deposited on glass slides and dried at room temperature. The images of AgNPs were obtained using a scanning electron microscope (KYKY, Model No. EM-3200).

### Evaluation of antibacterial effects of silver nanoparticles: Minimum inhibitory concentration.

*Y. ruckeri, V. cholerae* and *L. monocytogenes* bacteria were grown in nutrient broth (NB) medium. Concentrations of 10, 5, 2.5, 1.25, 0.62, 0.31 mg/mL synthesized silver nanoparticles were used to evaluate the antibacterial effects on *Y. ruckeri, V. cholerae* and *L. monocytogenes.* Concentrations were determined by broth micro-dilution technique in sterile 96 well plate. A volume of 100 μL of nanoparticles synthesized at concentration of 20 mg/mL was placed into the first well of the plate microtiter that contained 100 μL NB medium to obtain a concentration of 10 mg/mL. Serial dilution was performed by pumping the contents of the first well and removal of 100 μL from it and adding it to the second well. This operation was done to the last well. Then, 100 μL of bacterial suspension, equivalent to 0.5 mcFarland (1.5 × 10^6^ CFU/mL), was added to the wells. Each plate was prepared with a set of controls. Plates were placed in an incubator (Binder, USA) at 37°C for *V. cholerae* and *L. monocytogenes* and 30°C for *Y. ruckeri* for 18–24 hours. The lowest concentration at which no visible bacterial growth could be found was taken as the MIC value (21).

### Minimum bactericidal concentration.

The method used and described below is an amended version of the procedure described in the BSAC Guide to Sensitivity Testing and can be adapted for determining the minimum bactericidal concentration (MBC) of silver nanoparticles synthesized by biological method. After determining the MIC of silver nanoparticles synthesized, 10 μL from all wells that had no visible bacterial growth was removed and was then cultured on the NA media. Next, the plates were incubated for 1 night at 37°C for *V. cholerae* and *L. monocytogenes* and 30°C for *Y. ruckeri*. MBC is the lowest concentration of antimicrobial agent that openly kills >99.9% of the initial bacterial population where no visible growth of the bacteria is observed on NA medium (21).

### Antibacterial activity by agar well diffusion method.

Antibacterial activity of the synthesized AgNPs was analyzed using the agar well diffusion assay method. The 20 mL medium of mueller-hinton agar (MHA) semi-solid was poured into the petri dishes. The bacteria were grown in NB for 24 hours and by sterile swab on the surface of solid medium MHA and were cultured with 1.5 × 10^6^ CFU/mL suspensions of test bacteria. Different concentration of silver nanoparticles (ranging from 0.32 to 10 mg/mL) was impregnated into well (6 mm diameter) and placed on the surface of inoculated agar plates. Plates were incubated for 24 hours at 37°C for *V. cholerae* and *L. monocytogenes* and 30°C for *Y. ruckeri*. Antimicrobial activity was measured based on the diameter of inhibition zone in mm. Statistical analysis was performed using one-way analysis of variance with Tukey's test, which is used to compare the differences among samples. P values ≤ 0.05 were considered significant, and all antibacterial assays were performed with 3 replications.

## RESULTS

### AgNPs characterization: UV-vis spectra analysis.

**Fig. 1.**
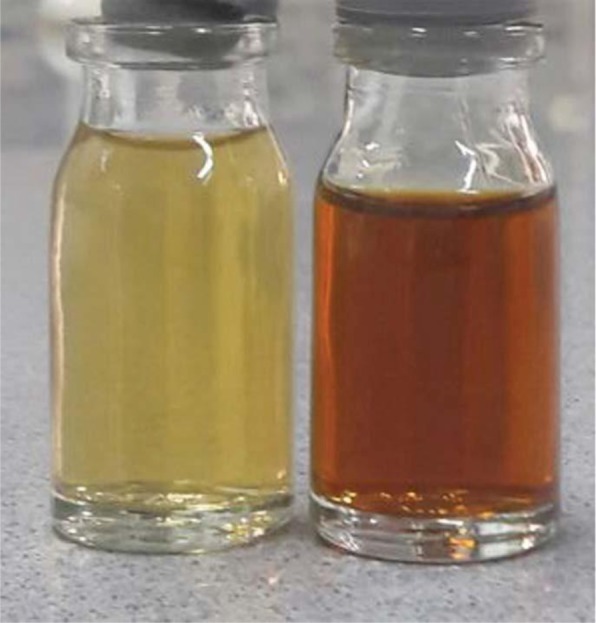
The color change of plant extract (left) before and (right) after adding a silver nitrate solution

**Fig. 2.**
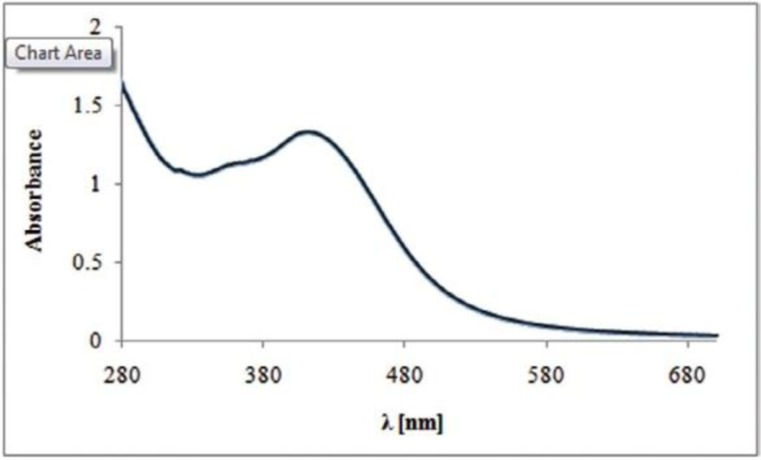
UV-Vis spectra of AgNPs biosynthesized from aqueous extract of *Verbena officinalis*

As the *V. officinalis* leaf aqueous extract was added to silver nitrate solution, the color of the solution changed from light yellow to reddish brown after the process of reduction of Ag+ to Ag nanoparticle, which indicated AgNPs formation (Fig. 1). Fig. 2 shows the UV–visible spectra of silver nanoparticle formation using constant AgNO _3_ concentration (2 mM) with *V. officinalis* leaf extract at 37°C after 24 hours between wavelength of 280 to 700 nm. The results of optical density showed that the maximum absorption measuring. solution containing the nanoparticles was at around 420 nm.

### Fourier transform infrared spectroscopy analysis.

**Fig. 3.**
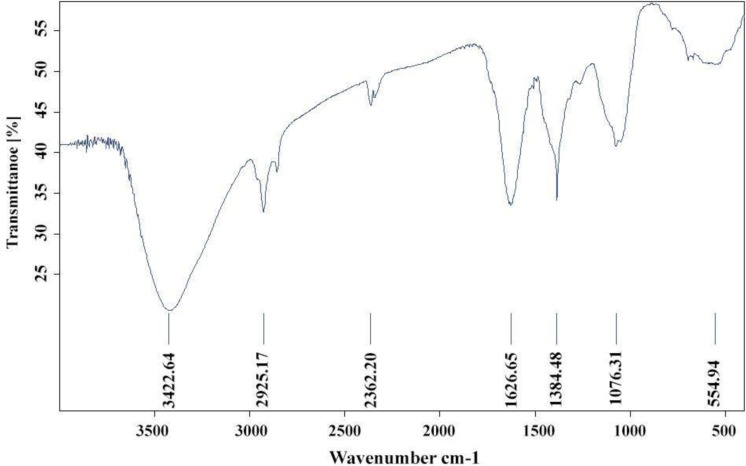
Graph obtained from FTIR analysis of AgNPs obtained From *V. officinalis* leaf extract

FTIR spectrum of Ag nanoparticles synthesized using *V. officinalis* leaf extract was shown in Fig. 3. Prominent bands of absorbance were observed at around 1075.31, 1384.48, 1626.65, 2925.17 and 3422.64 cm^−1^. The observed peaks at 1075.31 and 1384.48 cm^−1^ denote the stretching vibration of aliphatic and aromatic amines, respectively (22). Strong peak in 1626.65 was related to stretching the vibration (C = O) that usually exists in proteins and indicates the presence of protein in the plant extract as a reducing agent and a stabilizer (3). Relatively broad peak in 3422.64 cm^−1^ shows the presence of hydroxyl functional groups (O-H). These peaks show compounds of plant extract. Aliphatic C-H bonds, the intense peak in the range of 2850 to 3000 cm^−1^, and the presence of these peaks are observed in the frequency range of 2925.17 cm^−1^ in the structure of the plant extract.

### Scanning electron microscopy analysis.

**Fig. 4.**
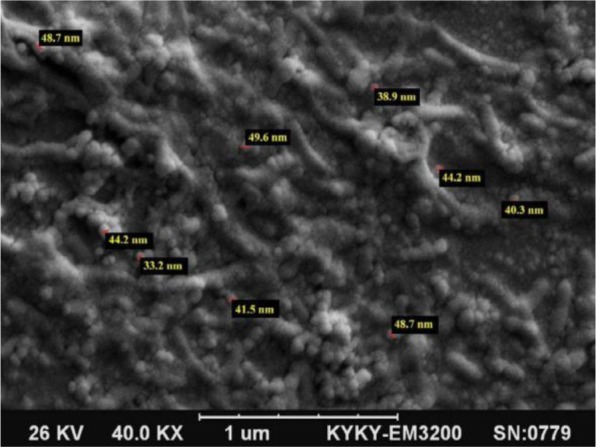
SEM images of silver nanoparticles formed by the reaction of 2 mm silver nitrate and 1 mL leaf extract of *V. officinalis*

The SEM image of the AgNPs is displayed in Fig. 4. Nanoparticles were formed with an average size of 42.57 ± 5.34 nm.

### Antibacterial property analysis.

In this study, the antimicrobial property of AgNPs was investigated by growing *Y. ruckeri, V. cholerae* and *L. monocytogenes* colonies on MHA plates, supplemented with different concentrations of AgNPs. Results obtained are shown in Table 1 and Fig. 7. The inhibition zones obtained indicate maximum antibacterial activity of the prepared test sample. The zone of bacterial inhibition by AgNPs prepared from *V. officinalis* leaf extract shows maximum inhibition for *Y. ruckeri* at 10 mg/mL, with 16 ± 0.5 mm and minimum inhibition for *L. monocytogenes* at 0.62 mg/mL, with 9.16 ± 0.28 mm. Also, in comparison with AgNPs, no antimicrobial activity was observed in the aqueous extract of plant (control).

**Table 1.** Inhibition zone diameter (mm) of AgNPs for *Listeria monocytogenes, Vibrio cholerae* and *Yersinia ruckeri* bacteria

**AgNPs (mg/mL)**	***Yersinia ruckeri***	***Vibrio cholerae***	***Listeria monocytogenes***
10	16 ± 0.5^a^	13.16 ± 0.28^a^	15.16 ± 0.57^a^
5	15.16 ± 0.28^a^	12.83 ± 0.28^ab^	14.66 ± 0.57^a^
2.5	14.5 ± 0.86^a^	12.16 ± 0.28^b^	13.33 ± 1.15^a^
1.25	11.66 ± 1.15^b^	10 ± 0.0^c^	10.83 ± 0.76^b^
0.62	10.66 ± 1.15^b^	9.66 ± 0.57^c^	9.16 ± 0.28^b^
Crude extract	0	0	0

### Minimum inhibitory concentration and minimum bactericidal concentration.

**Fig. 5.**
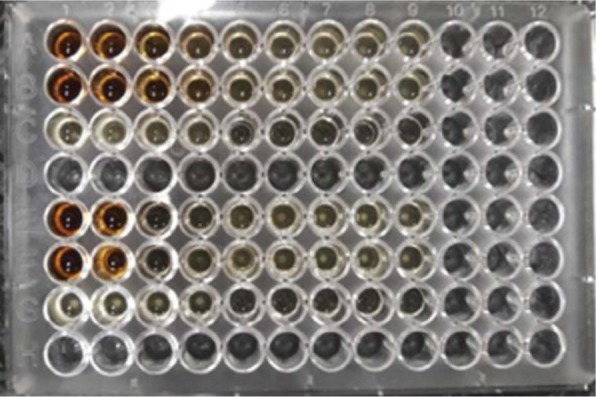
MIC of AgNPs synthesized with *V. officinalis* extract on the *L. monocytogenes* and *V. cholerae* bacteria in microtiter plate ( The top 2 rows (A1–A9 and B1–B9) was used for *L. monocytogenes*, and the bottom row (E1–E9 and F1–f9) for *V. cholerae*.

**Fig. 6.**
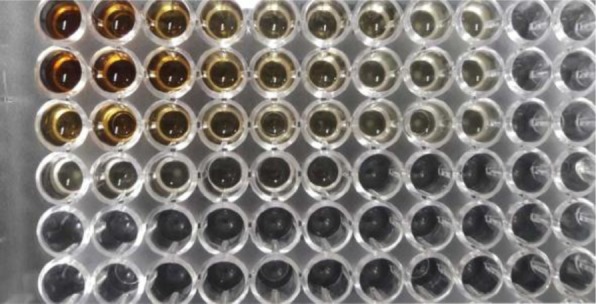
MIC of AgNPs synthesized by *V. officinalis* leaf extract on *Y. ruckeri*

The results of the MIC and MBC of nanoparticles synthesized on the *Y. ruckeri, V. cholerae* and *L. monocytogenes* bacteria are shown in Table 2 and Figs. 5 and 6. The values of the MIC and MBC of AgNPs were obtained to be 2.5 and 5 mg/mL for *V. cholerae*, 0.32 and 0.62 mg/mL for *Y. ruckeri*, 1.25 and 2.5 mg/mL for *L. monocytogenes*.

**Fig. 7.**
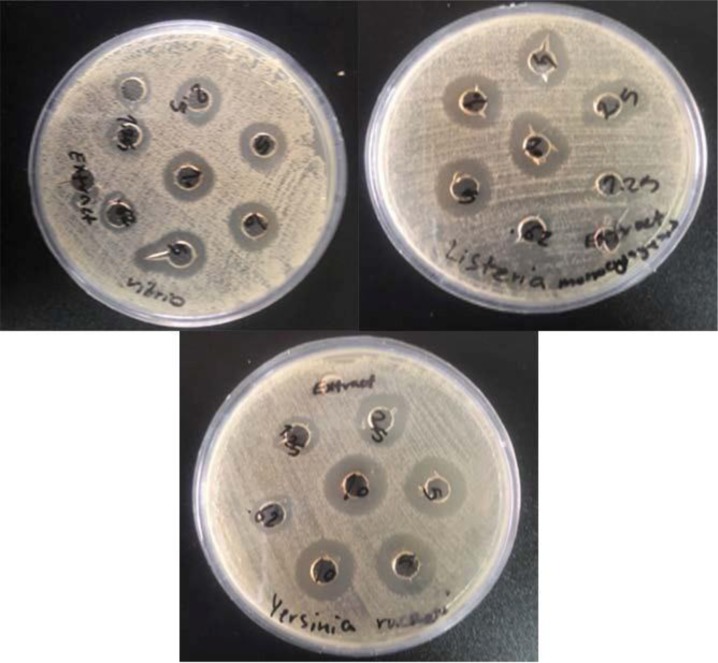
Inhibition zone diameter of concentration of 0.32–10 mg/mL AgNPs on (a) *V. cholerae*, (b) *Y. ruckeri*, and (c) *L. monocytogenes* bacteria

**Table 2.** The Minimum inhibitory concentration and minimum bactericidal concentration of AgNPs on the *Listeria monocytogenes, Vibrio cholerae* and *Yersinia ruckeri* bacteria

**AgNPs (mg/mL)**	**10**	**5**	**2.5**	**1.25**	**0.62**	**0.32**	**0.16**
*Yersinia ruckeri*	−	−	−	−	MBC	MIC	+
*Vibrio cholerae*	−	MBC	MIC	+	+	+	+
*Listeria monocytogenes*	−	−	MBC MIC		+	+	+

Growth of bacteria [+] and the inhibition of bacterial growth [−]

## DISCUSSION

Biological production of nanoparticles is a new, low-cost, low-risk method for producing nanoparticles that has caught the attention of scientists since 1990s (3). Silver nanoparticles, due to their small size, have unique physical and chemical properties. Nanoparticles have a more germicidal effect than the mass of silver metal because of the reduction of size, increase of the ratio of surface to volume of nanoparticles, and increase of the contact area with microorganisms (23).

The present study investigated the synthesis of nanoparticles and the methods used for the diagnosis and evaluation of antibacterial effects of nanoparticles by *V. officinalis* extract. In this study, after 24 hours of the conversion process, silver nanoparticle showed reddish-brown color, suggesting the formation of silver nanoparticles in solution and, in fact, confirmed the reaction between AgNO_3_ and *V. officinalis* leaf extract, which is similar to previous studies (4, 24, 25). In the present study, the size of silver nanoparticles synthesized using *V. officinalis* extract was obtained to be 33–49 nm. The synthesis of nanoparticles, with a size of 10–45 nm, using extracts of *Vitis vinifera*, was reported by Roy et al. in 2013 (26). In another study, silver nanoparticles, with an average size of 13–57 nm, were synthesized using extract *Ixora coccinea* (27). Green synthesized silver nanoparticles by *V. officinalis* leaf extract, with average size of 42.57 nm, were examined for their antibacterial activity using selected pathogens. In the present work, MHA well diffusion test was performed for antibacterial activity on Gram-positive bacteria, such as *L. monocytogenes* and Gram-negative bacteria, such as *Y. ruckeri* and *V. cholerae*. In the study of Mariselvam et al. (2014) (28), the zones were used to determine the activeness of the synthesized nanoparticles. The zone with the diameter of <9 mm was considered as inactive; 9–12 mm as partially active, and 13–18 mm as active. In this study, most of the AgNPs samples were active against *Y. ruckeri* and *L. monocytogenes* and less active against *V. cholerae*. In the present study, the effects of nanoparticles synthesized on Gram-positive and Gram-negative bacteria were almost identical. Jain et al. (2009) (29) studied the antibacterial effects of silver nanoparticles synthesized by papaya plant on *E. coli* (Gram-negative) and *Staphylococcus aureus* (Gram-positive) bacteria and their results were similar to those of the present study. The antimicrobial effects of silver nanoparticles on both Gram-positive and Gram-negative bacteria were evaluated and attributed to the same effects of silver nanoparticles on the wall and membrane of bacteria (30). Many sources have pointed to the deadly effects of silver nanoparticles due to simultaneous activity on the wall, the ability to penetrate the cell membrane, and the effect on cell respiration chain, RNA and DNA; these structures are the same in Gram-positive and Gram-negative. Thus, the antibacterial properties of silver nanoparticles in both groups of bacteria were approximately the same (31).

Our experiments revealed that there is maximum inhibition for *Y. ruckeri* at 10 mg/mL AgNPs, with 16 ± 0.5 mm, followed by *L. monocytogenes* (15.16 ± 0.57) and *V. cholerae* (13.16 ± 0.28). In a study, Kumarasamyraja and Jeganathan (2013) (20) showed that the maximum zone of inhibition at concentration of 300 μg/mL of *Acalypha indica* Ag-NPs, with average particle size of 0.516 μm on the *P. aeruginosa*, were 16 mm, followed by *E. coli* (14 mm) and *Bacillus subtilis* & *S. aureus* (13 mm); zone of growth inhibition in the face of nanoparticles synthesized by *A. indica* extract was almost the same as the zone of growth inhibition observed in this study. Also, their results indicated that *A. indica* AgNPs have potential antimicrobial activity against Gram-positive and Gram-negative bacteria, which is similar to the results observed in the present study. In the study of Rajesh et al. (2012) (32), antibacterial effects of biological synthesis of silver nanoparticles, using *Ulva fasciata* etyle acetat extract on *Xanthomonas Malvacearum*, showed inhibition zone diameter of 14±0.58 mm, which is similar to the results of the present study. In the study of Gnanadesigan et al. (2010) (23), the antibacterial potential of silver nanoparticle synthesized by *Avicennia marina* plant was evaluated, and the highest inhibition zone diameter was determined against *E. coli* with 18.40 ± 0.97 mm, and the minimum diameter zone of inhibition for *S. aureus* with 10.83 ± 1.33. Moreover, silver nanoparticles synthesized by *Ocimum tenuiflorum* extracts were found to have highest inhibition zone diameter (30 mm) against *S. aureus* and *E. coli* (33). Bactericidal activity by silver nanoparticles depends on the concentration of AgNO_3_. For example, if the metal concentration is lower, its antibacterial activity is more, and if the metal concentration is higher, its activity is less. The smaller particles have greater level of interaction, so they have more antibacterial effect compared to larger particles (34). The mechanism for the inhibition zone diameter may be due to the increase in proton motive force taking place on the surface of the bacteria due to ionic bond formation with AgNPs (35). Cell membranes of microorganisms are negatively charged and silver nanoparticles are positively charged; and when the charges of these particles come together, significant conformational changes are made in the membrane and the membrane eventually loses control of its permeability, leading to death of the cell (36). The MIC of the AgNPs sample, which effectively inhibits the growth of tested pathogens, is called MIC value of that particular sample. If the MIC of the sample is too low, the sample has strong antibacterial activity. In our study, the highest MIC and MBC of AgNPs (2.5 and 5 mg/mL) were observed for *V. cholerae*. The lowest MIC and MBC of AgNPs (0.32 and 0.62 mg/mL) were observed for *Y. ruckeri*, respectively. The MIC and MBC of AgNPs were found to be 1.25 and 2.5 mg/mL for L. monocytogenes. Silver nanoparticle synthesized by *Avicennia marina* plant against *E. coli* and *S. aureus* showed MIC and MBC in the range of 6.25 and 50 μg/mL (28). Silver nanoparticles synthesized using *Acalypha indica* leaf extract on *E. coli* and *V. cholerae* were investigated by Krishnaraj et al. (2010) (7). AgNPs with size of 20–30 nm showed MIC of 10 μg/mL. Saxena et al. (2010) (37) also studied the biosynthesis of silver nanoparticles by onion extract against *E. coli* and *Salmonella* Typhimurium; in their study, concentrations of 50 μg/mL confirmed an effective concentration. MIC in the above-mentioned studies was less than the value obtained in this study. Studies by Jain and Sharma (2013) also showed that it is possible to inhibit the growth of yeast at low concentrations of silver nanoparticles (30). Silver nanoparticles are most effective in preventing mitochondrial activity, and cell death occurs with the production of lytic enzymes. Nanoparticles show new or improved properties based on specific characteristics, such as size, distribution, and morphology (38). In addition to particle size, particle shape plays an important role in the antimicrobial properties of nanoparticle; form-less angular nanoparticles have the most properties and spherical and rod-shaped particles have the least properties, respectively (39). The low properties of this study may be due to the shape of the rod and the large particle size. The results clearly indicated that *V. officinalis* AgNPs have potential antimicrobial activity against Gram-positive and Gram-negative bacteria.
